# Walking along the Fibroblast Growth Factor 10 Route: A Key Pathway to Understand the Control and Regulation of Epithelial and Mesenchymal Cell-Lineage Formation during Lung Development and Repair after Injury

**DOI:** 10.1155/2014/538379

**Published:** 2014-09-11

**Authors:** Elie El Agha, Saverio Bellusci

**Affiliations:** ^1^Department of Internal Medicine II, Universities of Giessen and Marburg Lung Center (UGMLC), Klinikstraße 36, 35392 Giessen, Hessen, Germany; ^2^Member of the German Center for Lung Research (DZL), 35392 Giessen, Hessen, Germany; ^3^Developmental Biology and Regenerative Program of the Saban Research Institute at Childrens Hospital Los Angeles and University of Southern California, Los Angeles, CA 90027, USA

## Abstract

Basic research on embryonic lung development offers unique opportunities to make important discoveries that will impact human health. Developmental biologists interested in the molecular control of branching morphogenesis have intensively studied the developing lung, with its complex and seemingly stereotyped ramified structure. However, it is also an organ that is linked to a vast array of clinical problems in humans such as bronchopulmonary dysplasia in premature babies and emphysema, chronic obstructive pulmonary disease, fibrosis, and cancer in adults. Epithelial stem/progenitor cells reside in niches where they interact with specific extracellular matrices as well as with mesenchymal cells; the latter are still poorly characterized. Interactions of epithelial stem/progenitor cells with their microenvironments are usually instructive, controlling quiescence versus activation, proliferation, differentiation, and migration. During the past 18 years,* Fgf10* has emerged not only as a marker for the distal lung mesenchyme during early lung development, but also as a key player in branching morphogenesis and a critical component of the niche for epithelial stem cells. In this paper, we will present the current knowledge regarding the lineage tree in the lung, with special emphasis on cell-lineage decisions in the lung mesenchyme and the role of Fgf10 in this context.

## 1. Overview of Lung Development

**Figure 1:**
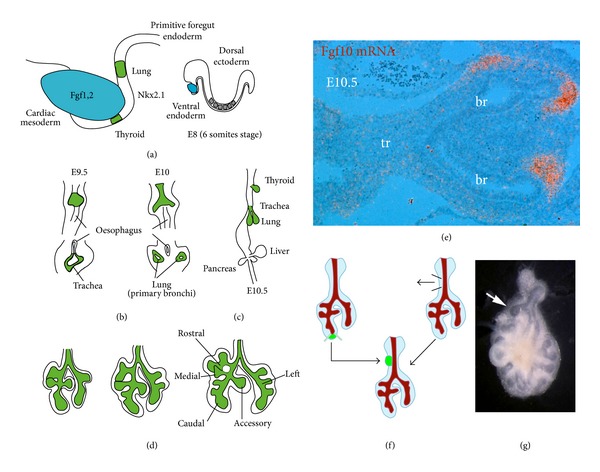
*Early mouse lung development*. (a) Interaction between the cardiac mesoderm, secreting Fgf1 and Fgf2, and the foregut endoderm specifies the* Nkx2.1*-positive territory (in green) from which the thyroid and the lung will form. (b, c) Emergence of the trachea on the ventral side of the foregut endoderm and of the two primitive bronchi. Lower panels in (b) are cross sections. (d) Through successive budding events, the right primitive bronchus gives rise to four lobes (rostral, medial, caudal, and accessory) while the left primitive bronchus gives rise to a unique left lobe. (e) Radioactive* in situ* hybridization on a section of E10.5 lung showing* Fgf10* mRNA expression (in red) in the distal mesenchyme. (f) Schematic of the experiment carried out by Alescio and Cassini [9]. The mesenchyme from the trachea is removed and replaced by distal lung mesenchyme. (g) The grafting of the distal mesenchyme in direct contact with the trachea induces the formation of an ectopic bud (arrow). Br: bronchus; tr: trachea.

During mouse embryogenesis, lung development starts at embryonic day 8 (E8) with the specification, in the primitive foregut endoderm, of the lung domain positive for the transcription factor* Nkx2.1* (also called thyroid transcription factor 1,* Ttf1*). This occurs through inductive signals involving Fgf1 and 2 arising from the cardiac mesoderm (Figure 1(a)). The respiratory tract per se starts to form at E9.5 as a budding of the ventral foregut endoderm allowing the formation of the future trachea ventrally and the esophagus dorsally (Figure 1(b)). Concomitantly, two buds, the future main bronchi, form distally of the tracheal tube (Figure 1(c)). These buds undergo iterated branching leading to the formation of one unique left lobe and four right lobes (rostral, medial, caudal, and accessory) in mice (Figure 1(d)). For more details on lung development, we refer the readers to excellent reviews previously published [1–5].

A major advance in our understanding of the branching process came with the characterization of fibroblast growth factor 10 (*Fgf10)* expression during early lung development [6].* Fgf10 *is an early marker of the lung mesenchyme expressed in the distal (submesothelial) mesenchyme (Figure 1(e)). It acts on the adjacent epithelium expressing fibroblast growth factor receptor 2-IIIb* (Fgfr2b) *to maintain the epithelial cells in a progenitor-like state and induce branching [6–8]. Interestingly, grafting of E12 distal lung mesenchymal domain expressing* Fgf10* at the level of the trachea, which normally does not undergo budding, is capable of inducing ectopic bud formation (Figures 1(f) and 1(g)) [9] and expression of the distal epithelial marker surfactant protein c (*Sftpc* or simply* Spc*) [10]. These results can be reproduced with recombinant Fgf10-coated heparin-sepharose beads [11] demonstrating that Fgf10 is sufficient to induce the budding process.

The exact mechanism of action of Fgf10 in controlling the branching process, more than 18 years after its discovery, is still unclear. Early transcriptional targets of Fgf10 during lung bud morphogenesis were investigated [7]. The authors used microarray analysis on mesenchyme-free lung epithelial explants and* in situ* hybridization on intact embryonic lungs to show that Fgf10 induces genes that are involved in cell rearrangement and migration, inflammatory processes, lipid metabolism, and tumor invasion. No proliferative effect on the lung epithelium was observed [7]. The consensus so far is that Fgf10 per se is acting primarily as a chemotactic factor for the lung epithelium. More recently, it has been suggested that Fgf10 controls the mitotic spindle orientation in the developing epithelium via Ras-regulated Erk1/2 signaling pathway, thus determining lung tube shape [12]. During murine lung development, Fgf10 functions in a dose-dependent manner as hypomorphic *Fgf*10^*lacZ*/−^ lungs, displaying around 80% decrease in* Fgf10* expression compared to* Fgf10* ^*+/+*^ lungs, reveal branching simplification and vascular abnormalities (demonstrated by the simplification of the vascular tree and the presence of large hemorrhagic areas). Decreased* Fgf10* expression in these lungs is associated with a decrease in the number of* Ttf1* and* Sftpb*-expressing cells, indicating that* Fgf10 *dosage is critical for the amplification of epithelial progenitors. Mesenchymal lineages, such as the endothelium and smooth muscle, are also affected in *Fgf*10^*lacZ*/−^ lungs as demonstrated by decreased numbers of* Pecam* and* αSma*-positive cells [13].* Fgf10* heterozygous mice are viable and reproduce normally even though they display impaired development of submandibular salivary glands, suggesting that* Fgf10* expression is required at different thresholds, in an organ-specific manner, to elicit normal development [14]. It remains to be established whether* Fgf10* heterozygous animals display the same repair response as wild-type animals following injury.

Considering the conservation of the initial branching pattern, Metzger et al. have proposed that the branching process is highly wired at the molecular level with the engagement of different routines and subroutines called domain branching, planar bifurcation, and orthogonal bifurcation [15]. More recently, Blanc et al. challenged this idea proposing that the developing lung epithelial tree adapts in real time to fill the available space in the mesenchyme, rather than being rigidly specified and predefined by a global genetic program [16]. Using a qualitative and quantitative* in vivo* morphometric analysis of the E11.25 to E13.5 mouse whole right cranial lobe structure, the authors show that beyond the first generations, the branching stereotypy relaxes and both spatial and temporal variations are common. The branching pattern and branching rate are sensitive to the dynamic changes of the mesoderm shape that is, in turn, mainly dependent upon the volume and shape of the surrounding intrathoracic organs. In addition, it has been proposed that it is the simple diffusion of Fgf10 from the distal mesenchyme that triggers differential epithelial proliferation, rather than a very sophisticated set of routines and subroutines that spontaneously lead to branching. Modeling* in silico* Fgf10 diffusion from submesothelial mesenchyme, where* Fgf10* is expressed, and computing epithelial and mesenchymal growth in a coupled manner, it was found that the resulting laplacian dynamics precisely account for the patterning of Fgf10-induced genes, and that the branching process spontaneously involves differential proliferation leading to a self-avoiding and space-filling tree[17, 18]. However, a limitation to this model, based on Fgf10 diffusion from a localized source, is that from E13.5 onwards,* Fgf10* becomes expressed throughout the mesenchyme [6] and a simple gradient of* Fgf10* expression is unlikely to explain the branching process, which is still actively underway. In addition, ubiquitous expression of* Fgf10* from the beginning of lung morphogenesis on the* Fgf10*-knockout background leads to the formation of apparently normal epithelial buds [19]. It is still unclear whether the stereotypic branching pattern is conserved in this model. It therefore seems that Fgf10 plays a permissive rather than an instructive role in the budding process.

**Figure 2:**
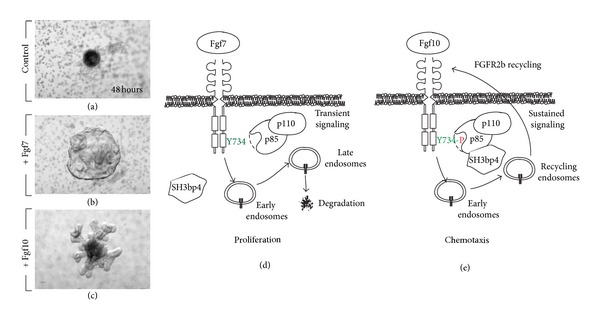
*Fgf7 and Fgf10 signaling via Fgfr2b controls proliferation versus chemotaxis, respectively*. (a)–(c) Mesenchyme-free epithelium from E11.5 lungs was grown for 48 hours in Matrigel in absence (a) or presence of Fgf7 (b) or Fgf10 (c). Note the formation of a cyst-like structure with Fgf7 versus distinct primary and secondary bud formation with Fgf10. (d), (e) Proposed model for Fgf7 versus Fgf10 action via Fgfr2b according to [20]. (d) Fgf7 induces transient signaling and Fgfr2b degradation leading to proliferation. (e) Fgf10 leads to sustained signaling which is associated with Fgfr2b recycling, phosphorylation of Y734 of Fgfr2b, and recruitment of p85/p110/SH3bp4 complex leading to chemotaxis.

We previously described that Fgf10 and Fgf7 ligands, both acting via Fgfr2b expressed in the epithelium, have different biological effects on isolated lung epithelium explants. Fgf7, which mostly triggers epithelial cell proliferation, induces the formation of a cyst-like structure Figure 2(b) while Fgf10, which acts mostly as a chemotactic/migration factor, induces the formation of multiple epithelial buds (Figure 2(c)). Using a mass spectrometry-based proteomics approach, we demonstrated how Fgf7 and Fgf10 lead to proliferation versus migration, respectively. While both ligands stimulated the phosphorylation of the autocatalytic tyrosine and the binding sites of Phospholipase C in Fgfr2b, Fgf10 differentially stimulated the phosphorylation of tyrosine 734 (Figures 2(d) and 2(e)) [20]. We showed that phosphorylation of this particular tyrosine residue controls the trafficking route of the receptor after internalization, allowing receptor recycling at the cell surface and sustained Akt and Shc phosphorylation. Phosphorylated Tyr734 interacts with the catalytic and regulatory domains of PI3 K. The regulatory domain of PI3 K contains an SH3 domain that binds SH3-domain-binding protein 4 (SH3bp4). Such interaction was detected in cells stimulated with Fgf10 but not with Fgf7. Disruption of this interaction prevented the previously described Fgf10-mediated trafficking of Fgfr2b to the recycling endosomes. Ectopic expression of a Y734F-mutated form of Fgfr2b or knockdown of* SH3bp4* in lung explants cultured* in vitro* changed the Fgf10 response from chemotactic (bud formation) to proliferative (cyst-like structure). It therefore appears that the chemotactic versus proliferative effects elicited by Fgf10 versus Fgf7 through Fgfr2b are linked to this differential phosphorylation of a single tyrosine residue of Fgfr2b (Figures 2(d) and 2(e)). Inducible* SH3bp4 *loss-of-function experiments and specific mutation of Y734 of Fgfr2b* in vivo* are currently underway to confirm these results in mice.

**Figure 3:**
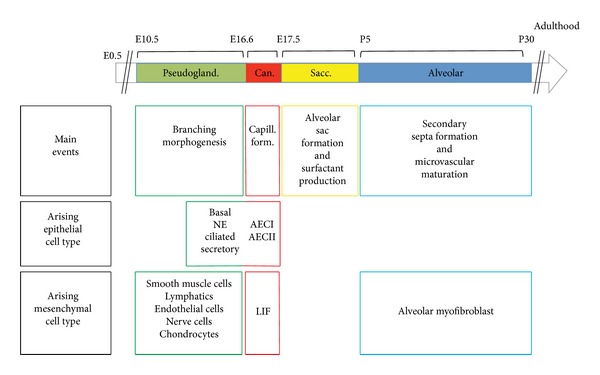
*Different phases of murine lung development*. Note that most epithelial and mesenchymal cell types are formed during the pseudoglandular stage of lung development. AEC: alveolar epithelial cells; Can: canalicular; LIF: lipofibroblasts; NE: neuroendocrine; Pseudogland: pseudoglandular; Sacc: saccular.

Lung development can be histologically divided into 4 stages. The first lung developmental stage is termed “Pseudoglandular stage” and it lasts until E16.5 (Figure 3). During this stage, many of the cell types that will populate the lung later on start to emerge. In the epithelium, these cells include basal, neuroendocrine, ciliated, and secretory cells. In the mesenchyme, the early pseudoglandular stage displays the formation of parabronchial smooth muscle cells (PBSMCs), vascular smooth muscle cells (VSMCs), endothelial cells, nerve cells, and cartilage and lymphatics. The lung then undergoes a short canalicular stage (E16.5 to E17.5) that is characterized by the formation of blood capillaries. During this stage, type I and type II alveolar epithelial cells (AECI and II) start to appear. In mice, functional alveoli start to form postnatally (from postnatal day 5 up to one month of age) whereas in humans, this process starts during late gestation and lasts up to two years. The alveolar stage is characterized by secondary septa formation with the appearance of alveolar myofibroblasts and microvascular maturation [21]. It is important to notice that most of the epithelial and mesenchymal cell types in the lung are forming during the second half of the pseudoglandular stage. Any developmental abnormality during that time period is therefore likely to result in impaired lung function at birth.

During the time course of lung development, proximal-distal patterning of the epithelial and mesenchymal cells arises from a complex network of signaling pathways that control the proliferation, migration, and differentiation of progenitor cells. In the next section, epithelial and mesenchymal progenitors during lung development will be reviewed.

### 1.1. Epithelial Progenitors Are Located at the Distal Tips in the Embryonic Lung

**Figure 4:**
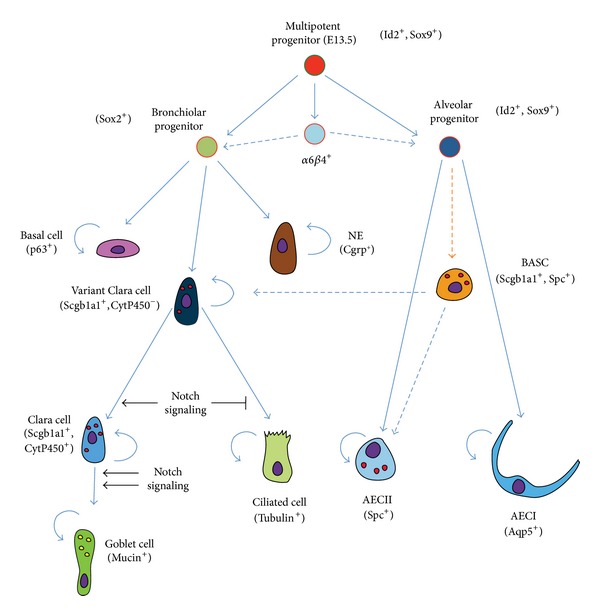
*Epithelial stem-cell tree during lung development*. The initial multipotent progenitor cells (Id2^+^, Sox9^+^), present throughout the lung epithelium up to E13.5, give rise to bronchiolar (proximal) and alveolar (distal) progenitors. As development proceeds, subsequent lineages are formed from these proximal (Sox2^+^) or distal (Id2^+^, Sox9^+^) epithelial progenitors. Most of the resulting cell types (such as p63^+^ basal cells, neuroendocrine cells, AECI, AECII, ciliated cells, and goblet cells) are unipotent. Clara cell progenitors (variant Clara cells) are at least bipotent as they can give rise to the secretory and ciliated lineages. These cells can be distinguished from mature Clara cells by the lack of cytochrome P450 expression. We also propose that the *α*6*β*4-double positive cells and the BASCs (Scgb1a1^+^, Sftpc^+^) are formed during lung development. The origin of these cells is still unclear (orange dashed lines). Their respective contribution to the proximal or distal epithelial lineages during normal development is likely minimal (blue dashed lines). BASC: bronchoalveolar stem cell.

The epithelial stem-cell tree as it is currently understood is presented in Figure 4. One of the early attempts to establish an epithelial progenitor-cell hierarchy in the embryonic lung was done by Wuenschell et al. who used an immunohistochemical approach to demonstrate the spatiotemporal expression of epithelial markers during development [22]. The authors used calcitonin gene-related peptide (Cgrp), Clara cell secretory protein (Ccsp or Scgb1a1), and surfactant protein a (Sftpa) as markers of differentiated neuroendocrine cells, Clara cells, and AECII, respectively. The authors concluded that all three markers are coexpressed in the distal epithelium between E13 and E15, after which they are restricted to specific cell lineages. The data suggest that most epithelial lineages are derived from common progenitors from the pseudoglandular stage. These multipotent progenitors are identified on the basis of* Sox9* and inhibitor of differentiation 2* (Id2) *expression. As lung development progresses, the distinction between “proximal” bronchiolar progenitor cells (*Sox2*-positive) and distal “alveolar” progenitor cells (positive for* Sox9* and* Id2*) can be made.

More recently, Rawlins et al. used an* Id2* ^*Cre-ERT2*^ knock-in mouse line to target epithelial progenitors during the pseudoglandular and canalicular stages of lung development [23]. Their data show that* Id2*-positive progenitor cells, located in the distal tips of the lung epithelium, give rise to all epithelial cell lineages (bronchiolar and alveolar lineages) when labeled at the pseudoglandular stage. In contrast, these cells only contribute to alveolar cells (AECI and II) when labeled at the canalicular stage. Postnatally,* Id2* expression does not seem to identify a population of epithelial progenitors.

On the other hand, Perl et al. used transgenic mice carrying (rat)* Ccsp-rtTA*;* tet(O)Cre *constructs combined with Alkaline phosphatase or GFP reporters to demonstrate that ciliated and secretory (Clara and goblet cells), but not neuroendocrine cells, derive from Scgb1a1^+^ progenitors [24]. What controls the transition from a branching program to an alveolar program at the level of the distal epithelial progenitor cells is still unclear. It was recently shown that the forced activation of Kras in the distal epithelium suppresses the alveolar differentiation program [25]. The maintenance of the distal alveolar progenitor cell status is likely under the control of Fgf10 signaling. Continuous overexpression of* Fgf10* during lung development prevents the formation of* Sox2*-positive bronchiolar progenitor cells [19]. Such overexpression also prevents the differentiation of already committed* Sox2*-positive bronchiolar progenitor cells to the ciliated-cell lineage but facilitates their commitment to the p63^+^ basal-cell lineage.

In this context, Notch signaling pathway has been proposed to be critical for establishing a balance between ciliated and nonciliated/secretory cell fates in the developing lung. Conditional inactivation of Notch signaling in the endoderm using *Shh* ^*Cre*^ leads to the enrichment of ciliated cells at the expense of nonciliated secretory cells [26]. Moreover, members of the Forkhead box protein family have also been proposed to be involved in specifying cell fate and controlling proliferation rates of epithelial progenitors. Recently, it has been shown that FoxP1 and 4 transcription factors act during development to repress the goblet cell program in epithelial progenitors [27]. Prenatally, different levels of Notch activation influence cell fate of bronchiolar progenitors. In undifferentiated bronchiolar progenitors, lack of Notch signaling favors the ciliated and neuroendocrine cell fate, whereas normal or exacerbated levels of Notch signaling lead to differentiation into Clara or goblet cell fates, respectively. Once bronchiolar progenitors are committed to the Clara cell fate, Notch signaling is required to restrict a goblet cell differentiation program in a subpopulation of Clara cells during the postnatal period. Notch signaling could also be instrumental in maintaining the balance between ciliated and secretory cells in adult airways [28].

Wnt signaling has been shown to play an important role in proximal-distal patterning of the developing lung epithelium. Loss of function of *β*-Catenin signaling using* Spc-rtTA; tet(O)Cre* system leads to the inhibition of distal airway formation and enlargement of the proximal airways [29]. An opposite phenotype was reported with gain of function of *β*-Catenin signaling using* Ccsp-rtTA; tet(O)Cre* system [30].

A final type of epithelial progenitors is represented by the α6*β*4-double positive cells, which have been shown to give rise to both alveolar and bronchiolar cells [31, 32]. While these cells can be isolated in the adult lung, it is still unclear when these cells form during embryonic lung development and from which progenitor(s) they originate.

To sum up, distal epithelial progenitors, having a high proliferation index and expressing* Sox9* and* Id2*, give rise to most of the epithelial cell types during the pseudoglandular stage of lung development. As these cells lose* Sox9* and* Id2* expression and start expressing the proximal lung marker* Sox2*, their lineage commitment becomes restricted to specific cell types. Notch, Wnt, Bmp, and Fgf signaling pathways are believed to be involved in fate determination of epithelial progenitors during lung development (Reviewed in [4, 5]).

### 1.2. Mesenchymal Progenitor Cells in the Embryonic Mouse Lung

The lung mesenchyme consists of a wide array of cell types such as SMCs, endothelial cells, chondrocytes, nerve cells, lipofibroblasts, alveolar myofibroblasts, lymphatic cells, and others that play crucial roles during development and homeostasis after birth. Some of these cell types derive from common multipotent mesenchymal progenitors whereas others derive from unique progenitor populations.

**Figure 5:**
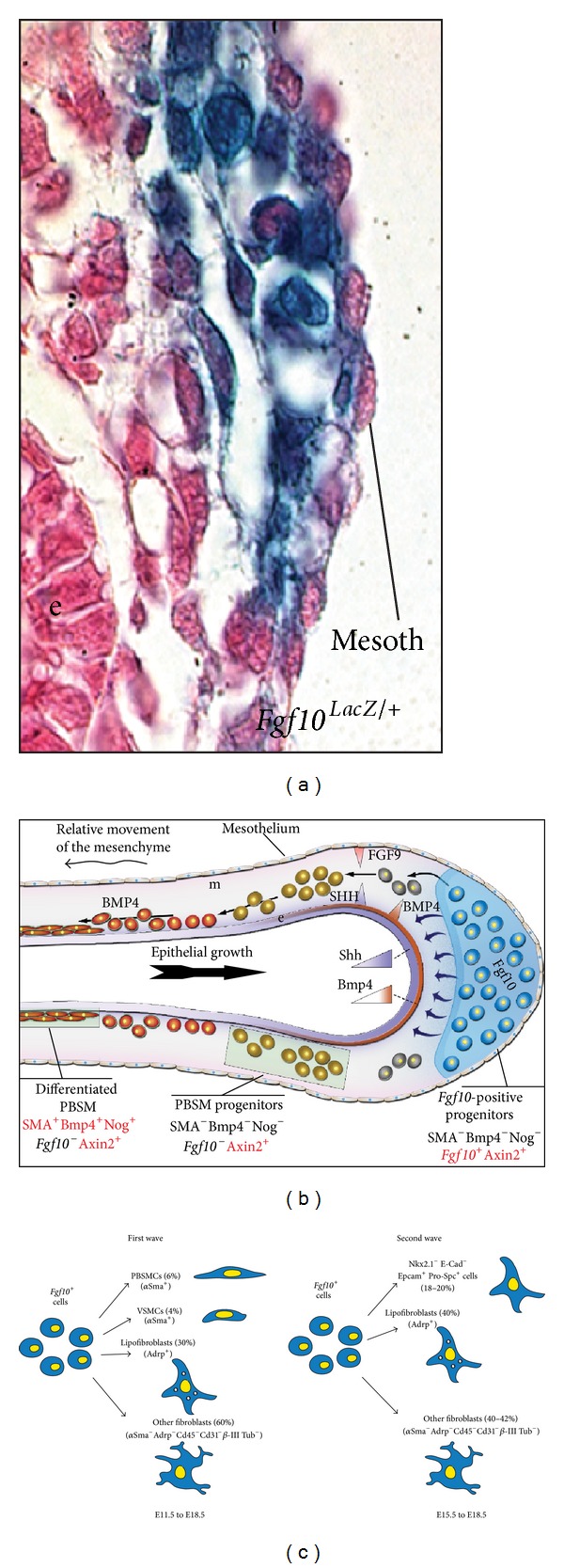
*Fgf10*-*positive cells represent a population of mesenchymal progenitors*. (a)* Fgf10* ^*lacZ*^ cells at E12.5 are located in the distal lung mesenchyme. (b) Model for the formation of the parabronchial smooth muscle cells.* Fgf10*-positive cells are amplified and then they differentiate as they relocate proximally (From [33]). (c) Lineage tracing of* Fgf10*-positive cells at E11.5 and E15.5 show that these cells are progenitors for multiple lineages and they become progressively restricted in their differentiation potential (From [34]).

A number of mesenchymal progenitors have been identified using transgenic and knock-in mouse lines that allow fate mapping of mesenchymal cells during lung development. We have previously utilized the enhancer trap transgenic line* Mlcv1v-nLacZ-24 *(or simply* Fgf10* ^*lacZ*^) to show that* Fgf10*-positive cells serve as progenitors for PBSMCs in the distal lung during early lung development (Figures 5(a) and 5(b)) [33]. We recently confirmed these results using an* Fgf10* ^*Cre-ERT2*^ knock-in line (Figure 5(c)) [34]. Bmp4, a downstream target of Fgf10 in the epithelium, appears to control the differentiation of* Fgf10*-positive cells along the PBSMC lineage [33]. At the early pseudoglandular stage, Fgf10 itself is not acting on the PBSMCs to control their differentiation.

As a follow-up on this finding, we have reported that *β*-Catenin signaling in the mesenchyme, mediated by the Pitx family of transcription factors, is critical for the amplification but not the differentiation of* Fgf10*-expressing progenitor cells [35]. We also recently showed that miR142-3p, a microRNA that is differentially expressed in the mesenchyme, controls the expression of adenomatous polyposis coli (*Apc*), a negative regulator of *β*-Catenin. Increased* Apc* expression upon miR142 attenuation leads to arrested mesenchymal proliferation and premature SMC differentiation [36]. It has also been reported that a population of mesenchymal cells, initially located near the lung hilum, could also serve as progenitors for PBSMCs [37].

We have previously generated an* Fgf10* ^*Cre-ERT2*^ knock-in mouse line that allows lineage tracing of* Fgf10*-positive cells postnatally and during development [38]. Lineage-tracing experiments, using these mice, revealed the presence of two waves of* Fgf10* expression during embryonic lung development: an early wave, from early pseudoglandular stage, consisting of cells (and their progeny) enriched at the periphery of the lung at E18.5; and a late wave, from late pseudoglandular stage, consisting of cells (and their progeny) dispersed throughout the lung mesenchyme at E18.5. These cells mainly differentiate along the airway and vascular SMC lineages (early wave only) and the lipofibroblast lineage (for both waves). We also identified an interesting mesenchymal Nkx2.1^−^, E-Cad^−^, Epcam^+^, and Pro-Spc^+^ lineage that arises from the late wave and seems to transiently express* Fgf10*. The significance of the latter cells is still unclear. Taken together, we showed that* Fgf10*-expressing cells represent a pool of mesenchymal progenitors for multiple lineages in the embryonic lung (Figure 5(c)) [34]. It is still unclear whether this population contains unipotent or multipotent progenitor cells. Analysis of the progeny of* Fgf10*-expressing cells using the multicolor-reporter Confetti mice needs to be carried out to better characterize these progenitor cells.

**Figure 6:**
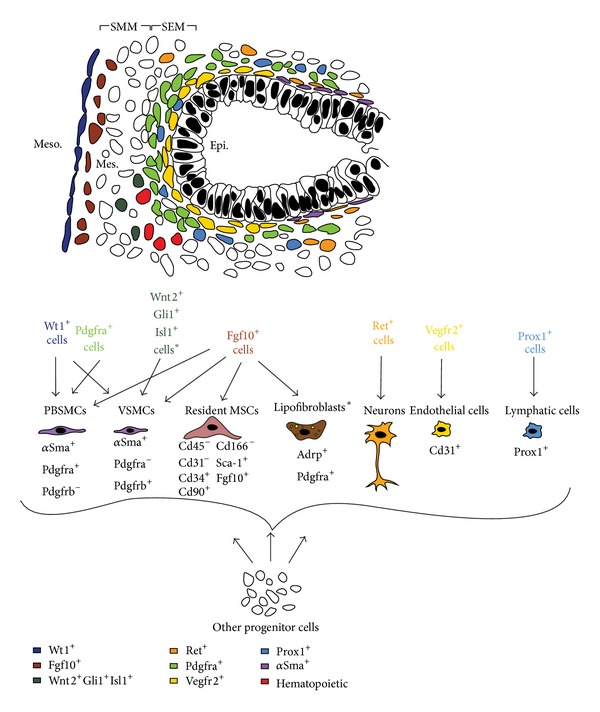
*Formation of the different mesenchymal populations during lung development*. (a) Schematic of the cells in the distal part of the lung at E13.5 showing the different lung domains including the mesothelium (meso), the submesothelial mesenchyme (SMM), the subepithelial mesenchyme (SEM), and the epithelium (epi). (b) Position of the different mesenchymal cell progenitors at this stage.

As shown in Figure 6, the earliest and simplest developmental stage that allows studying the localization and formation of multiple mesenchymal cell types in the embryonic lung is the midpseudoglandular stage (around E13.5). At this stage, it is easy to distinguish the three layers that constitute the lung as follows: the mesothelium, which constitutes the outermost layer and the mesenchyme and the epithelium, which is the innermost layer. The mesenchyme can be further divided into two compartments, each of which is populated by distinct mesenchymal progenitors: the dense circumferentially-oriented subepithelial mesenchyme (SEM) and the rather loose nonoriented submesothelial mesenchyme (SMM) [39] (Figure 6(a)). Figure 6 summarizes the localization of the different types of mesenchymal progenitors in the distal lung and our actual understanding of lineage formation in the mesenchyme during lung development. Que et al. utilized Wilms tumor 1 homolog* (Wt1)-Cre* transgenic line and suggested that the mesothelium contains vascular—but not airway—smooth muscle progenitors [40]. On the other hand, fate-mapping of* Pdgfrb*-positive cells showed that VSMCs arise from mesenchymal rather than mesothelial progenitor cells [41]. More recently, it was shown, using* Wt1* ^*Cre-ERT2/+*^ knock-in mice, that the mesothelium is a source of airway and vascular SMCs and desmin^+^ fibroblasts [42]. Endothelial progenitors, on the other hand, are characterized by the expression of fetal liver kinase 1* (Flk-1* or* Vegfr2)* [43–46] whereas lymphatic cells arise from prospero homeobox protein 1* (Prox1)*-positive progenitor cells [47]. Finally, nerve cells are believed to originate from the neural crest and they are marked by receptor tyrosine kinase* (Ret)* expression [48]. It was recently reported that a multipotent cardiopulmonary progenitor cell population (Wnt2^+^, Gli1^+^, Isl1^+^), originating from the second heart field (SHF), invades the lung, giving rise to airway and vascular SMCs in addition to other lineages [49].

### 1.3. Interactions between the Different Lung Domains Are Critical for Harmonious Lung Formation

It is widely accepted that the interaction between the aforementioned cellular domains is critical for the amplification of progenitors and cell-fate determination. Tissue interactions and their role in lineage commitment of mesenchymal cells will be discussed in the following section.

#### 1.3.1. Mesothelial-Mesenchymal Interactions: The SMM Is under the Effect of Fgf9 Signaling via the Wnt Pathway

**Figure 7:**
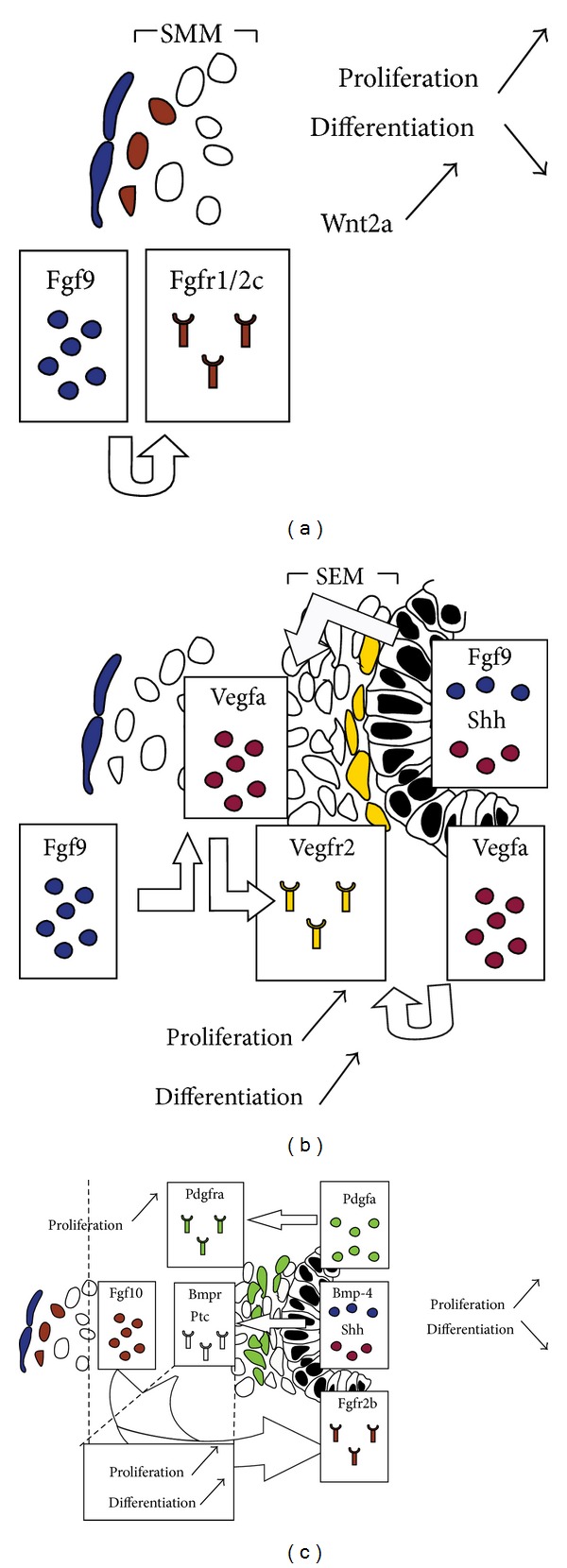
*Interactions between the different lung domains*. (a) Mesothelial-SMM interactions. (b) Epithelial-SEM interactions (mostly endothelial) and mesothelial-SEM interactions. (c) Epithelial-mesenchymal (SEM+SMM) interactions.

Both being of mesodermal origin, the lung mesothelium and the adjacent mesenchyme undergo paracrine signaling that is critical for proper lung development. Fgf9, secreted by the mesothelium, acts through the mesenchymal splice variant (c) of fibroblast growth factor receptors 1 and 2 (Fgfr1/2-IIIc) to induce proliferation of mesenchymal cells in the SMM, including* Fgf10*-positive cells [50] (Figure 7(a)). Fgf9 maintains these cells in a progenitor-like state by preventing their differentiation to SMCs. Loss of function of*β* *-Catenin* in the lung mesenchyme using* Dermo1* ^*Cre*^; *β* *-Catenin* ^*flox *^system has shown that the activity of Fgf9 on mesenchymal cells is mediated by Wnt signaling. Wnt2a seems to provide a permissive signal for Fgf9 signaling by controlling the expression of* Fgfr2c*. In turn, Fgf9 acts via Fgfr2c, to control the proliferation of mesenchymal cells in the SMM [35, 39, 43, 44, 51–53]. Furthermore, del Moral et al. have shown that Fgf9 acts not only on the mesenchyme, but also on the epithelium to induce proliferation, but not differentiation, via Wnt signaling [43, 44].

#### 1.3.2. Endothelial-Epithelial Interactions: Vegfa/Vegfr2 Signaling Is Critical for the Formation of the Capillary Network

The lung vasculature starts to develop as soon as the primary lung buds are formed [54]. DeMello and colleagues proposed a model in which central vessels (proximal) are formed by angiogenesis (branching of preexisting vessels) and peripheral vessels (distal) by vasculogenesis (*in situ* differentiation of endothelial progenitors). The two components fuse to form a continuous vascular lumen via a lytic process around E13/14 and the pulmonary circulation would be established [55].

The earliest marker of endothelial cells in the developing lung is* Vegfr2*. Vegfa, an endothelial cell mitogen and angiogenic factor, is secreted by the developing epithelium and mesenchyme and it acts on* Vegfr2*-positive progenitor cells residing in the SEM [43, 44, 56] (Figure 7(b)). These mesenchymal cells eventually give rise to the capillary plexus that surrounds the epithelium. White et al. have shown that Shh (epithelium-derived) and Fgf9 (epithelium and mesothelium-derived) upregulate the expression of* Vegfa* in the lung mesenchyme (Figure 7(b)) [57]. On the other hand, Vegfa has also been shown to act indirectly on the lung epithelium. del Moral et al. have shown that exogenous Vegfa promotes branching morphogenesis in the epithelium and increases the proliferation index in both the epithelium and mesenchyme of E11 lung explants. Vegfa upregulates* Bmp4* and* Sftpc* and induces proliferation of* Vegfr2*-positive cells [43, 44].

Endothelial-epithelial interactions have been shown to be critical for lung regeneration after pneumonectomy. Ding et al. have demonstrated that Vegfr2 and Fgfr1 activation in pulmonary capillary endothelial cells is required for inducing Mmp14 secretion. Mmp14 unmasks Egfr ectodomains in the lung epithelium and thus increases the bioavailability of Egfr ligands. This mechanism induces the expansion of epithelial progenitors during compensatory lung growth [58].

#### 1.3.3. Epithelial-Mesenchymal Interactions: Paracrine Signaling Controls Cell Proliferation and Differentiation in the Epithelium and Mesenchyme

Epithelial-mesenchymal interactions are critical for proper lung development. Shh, Bmp4, Fgf, and Wnt ligands are diffusible molecules that mediate epithelial-mesenchymal interactions in the developing lung (Reviewed in [59]). Shh, secreted by the epithelium, acts through its mesenchymal receptor Patched (Ptc) to induce mesenchymal cell proliferation and differentiation (Figure 7(c)). Treatment of E11.5 lung explants with recombinant Shh has shown to upregulate mesenchymal markers including its own receptors (*Ptc)*,* Noggin*,* Sma*, and* Myosin *[60, 61]. On the other hand,* Bmp4* has a dynamic expression pattern in the distal endoderm and the mesenchyme during early pseudoglandular stage (E11.5). Its expression is rather restricted to the epithelium beyond E13.5 (Figure 7(c)). Bmp4 mediates epithelial-interactions and is critical for PBSMC formation [33, 61, 62].

One of the main features of Shh and Bmp4 signaling is inhibition of* Fgf10* expression and activity, respectively. As already mentioned, Fgf10 is one of the key growth factors secreted by stromal cells in the distal mesenchyme [6]. It acts in a paracrine fashion on the opposite epithelium expressing Fgfr2b (Figure 7(c)). Fgf10/Fgfr2b signaling is critical for proper lung development and disruption of this pathway by genetic manipulation leads to detrimental consequences that range from branching simplification to agenesis of the lung [63–66]. Fgf10 promotes epithelial survival and branching and its dosage is crucial for the amplification of epithelial progenitors [13].

### 1.4. Mesenchymal Progenitor Cells in the Neonatal Mouse Lung

In mice, the last stage of lung development occurs postnatally and is characterized by the transition from primitive alveoli (sacs) to mature alveoli (Figure 3). This stage is characterized by the prevalence of two mesenchymal lineages: alveolar myofibroblasts (MYF) and lipofibroblasts (LIF).

#### 1.4.1. Alveolar Myofibroblasts

Alveolar MYF represent a population of* αSma*-positive interstitial fibroblasts that populate the lung during postnatal alveolarization. These cells deposit ECM fibers such as elastin and collagen that are critical for secondary-septa formation [67–69] and are believed to derive from* Pdgfra*-positive cells. In support of this possibility,* Pdgfa*-null newborns suffer from the absence of alveolar MYF and consequently arrest in alveologenesis [70, 71]. Lineage tracing of* Pdgfra*-positive cells during development is however still lacking and it is not demonstrated so far that* Pdgfra*-positive cells at the pseudoglandular stage are progenitors for alveolar MYF. Postnatally,* Pdgfra *is expressed by multiple mesenchymal cell types including PBSMCs, alveolar MYF, and LIF. Interestingly, forced Fgf activation in the lung mesenchyme, following the formation of an ectopic mesenchymal Fgf10-Fgfr2b autocrine loop from the early pseudoglandular stage, also leads to the absence of alveolar MYF formation at birth [51]. This would suggest that Fgf signaling represses the differentiation of the early alveolar MYF progenitors. It is not clear if this is due to a direct effect of Fgf on the mesenchyme or to an indirect effect via the epithelium. However, Fgf9, which is the natural and main Fgf ligand acting on the mesenchyme during early lung development, is capable of repressing the expression of* αSma* in primary cultures of lung mesenchymal cells, suggesting that Fgf signaling in the mesenchyme represses* αSma* expression, thus maintaining the mesenchymal progenitors undifferentiated and proliferative. A counter-intuitive result was obtained by Perl and Gale. The authors took advantage of the* Spc-rtTA/+; tet(O)solFgfr2b/+* double-transgenic mice to induce, in the lung, the expression of a soluble form of Fgfr2b acting as a decoy receptor for all Fgfr2b ligands. Expression of this decoy receptor in the lung from E14.5 to E18.5 disrupts alveologenesis postnatally. Secondary-septa formation with presence of alveolar MYF can be partially enhanced in this model by treating the animals with retinoic acid (RA), a vitamin A derivative. The effect of RA can be blocked by the reexpression of the soluble form of Fgfr2b. This leads to an increase in* Pdgfra*-positive cells and an associated decrease in* αSma*-positive cells. It was proposed that these* Pdgfra*-positive cells giving rise to alveolar MYF were LIF. This work also suggested that an Fgfr2b ligand, which is likely Fgf10, was directly or indirectly involved in the differentiation of these* Pdgfra*-positive cells into alveolar MYF [64]. As a follow up on this work, Fgfr2b ligands are required for alveolar MYF formation during alveolar regeneration after pneumonectomy [72]. However, the attenuation of all Fgfr2b ligands postnatally does not result in any lung development defect. Alveologenesis, which is characterized by the formation of secondary septa containing alveolar MYF, occurs normally. It can therefore be concluded that normal alveologenesis does not require secreted Fgfr2b ligands.

#### 1.4.2. Lipofibroblasts

LIF are adipocyte-like interstitial fibroblasts that reside in the late fetal and postnatal lung parenchyma in the immediate vicinity of AECII [68, 73]. It has recently been proposed that LIF constitute a stem-cell niche for AECII stem cells. LIF support the growth of AECII cells grown in Matrigel to form alveolosphere-like structures [74]. How LIF support the growth of AECII cells is still unknown. We have recently shown that some of the LIF derive from* Fgf10*-positive cells [34] and that a significant proportion of the LIF in the postnatal lungs express* Fgf10* (Al Alam, El Agha, and Bellusci, manuscript in preparation). It is therefore tempting to speculate that Fgf10, similarly to its function during development, is involved in the maintenance of the AECII stem cells in the adult lung during homeostasis and/or after injury. More research will have to be done to specifically identify the role of Fgf10 in LIF formation/maintenance in the context of the adult AECII stem-cell niche. Among the unanswered questions are the molecular and cellular differences between the diverse subpopulations, the* Fgf10*-positive and the* Fgf10*-negative, forming the LIF, and the mechanisms controlling the formation of the LIF during development. Interestingly,* Fgf10* expression is induced in NIH3T3-L1 preadipogenic cells by adipose differentiation medium (containing insulin and cortisone). Fgf10 activity is required for the differentiation of the preadipocytes into mature adipocytes [75]. In the future, it will be interesting to examine the effect of insulin and Fgf10 signaling on LIF progenitors to control their commitment along the LIF lineage. AECII signal to LIF via parathyroid hormone-related protein (Pthrp) to activate the peroxisome proliferator-activated receptor gamma (Ppar*γ*) pathway. This signaling pathway appears to be critical for the induction and maintenance of the LIF phenotype.* In vivo* inactivation of the Pthrp pathway leads to abnormal alveolarization with defective surfactant synthesis [76–78]. In response to Ppar*γ* activation, LIF express adipose differentiation-related protein (*Adrp*) that is critical for trafficking of lipid substrates and subsequently providing AECII with triglycerides for surfactant production [79]. LIF also secrete leptin that stimulates surfactant production by AECII [80]. In addition to triglycerides, LIF also store RAs that are essential for alveolar septation [81].

## 2. Progenitor/Stem Cells in the Adult Mouse Lung

**Figure 8:**
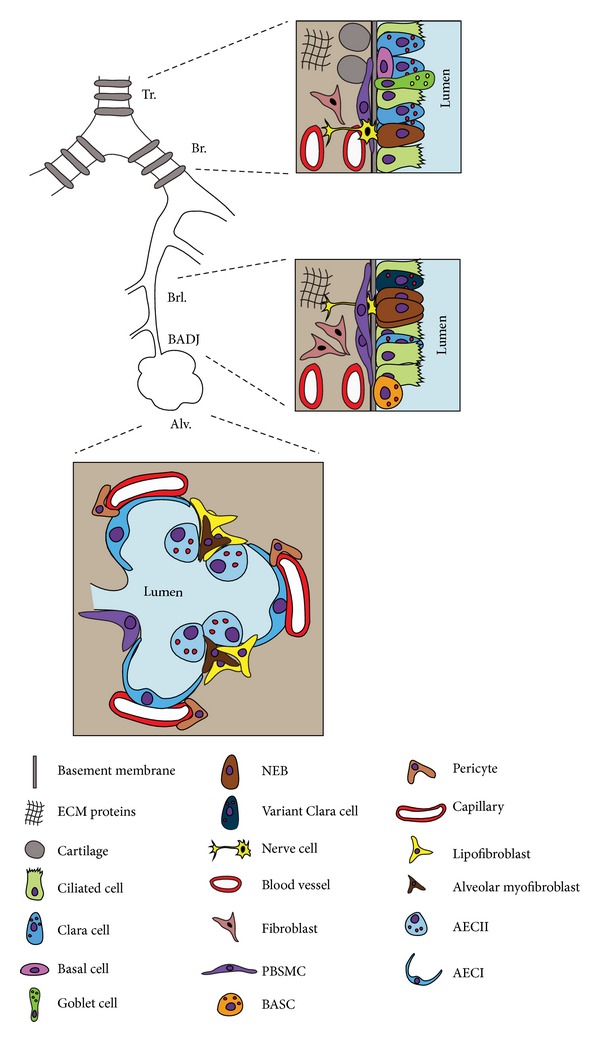
*Epithelial and mesenchymal cell diversity in the adult mouse lung*. The adult mouse lung can be subdivided into proximal (trachea, mainstem bronchi, and conducting airways) and distal (alveoli) domains. Each domain contains a specific set of epithelial and mesenchymal cells. NEB: neuroepithelial body.

The adult mouse lung consists of at least 40–60 different types of cells that are organized in a highly sophisticated 3D structure inside the thoracic cavity [82, 83]. From an anatomical point of view, the mouse lung can be divided into proximal and distal regions (Figure 8). The proximal region consists of the trachea, mainstem bronchi, and conducting airways and is populated by basal cells, goblet cells, neuroendocrine cells, ciliated cells, and nonciliated secretory cells. In mice, cartilage rings cover the trachea and mainstem bronchi whereas in humans, these rings are found deep in the lung. On the other hand, the distal region of the mouse lung is characterized by the absence of basal cells or cartilage tissue. This region contains few goblet cells and in addition to ciliated and secretory cells, AECI and II. AECI are squamous epithelial cells that occupy around 95% of the alveolar space and are responsible for gas exchange. AECII are cuboidal epithelial cells and they produce surfactants that maintain a low surface tension in the alveolar space and prevent the alveoli from collapsing (Reviewed in [4, 83]). In the mesenchymal compartment, SMCs, endothelial cells, chondrocytes, nerve cells, lipofibroblasts, myofibroblasts, lymphatic cells, and others can be found. Along the proximo-distal, the mouse lung is rich with stem/progenitor cells, in both the epithelial and mesenchymal compartments, that are capable of self-renewal, multipotent or lineage-restricted differentiation, and pollutant resistance.

### 2.1. Epithelial Progenitor Cells and Their Niches in the Adult Mouse Lung

**Figure 9:**
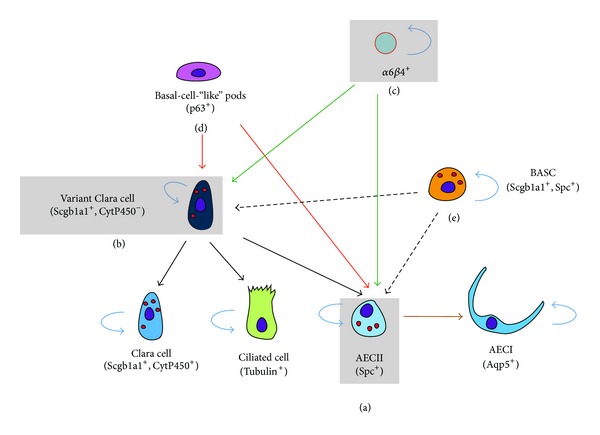
*Epithelial progenitor cells in the adult lung after injury*. (a) AECII serve as progenitors for AECI and they self-renew after hyperoxic injury. (b) In case of more pronounced injury, variant Clara cells serve as progenitors for the secretory and ciliated lineages as well as AECII. More recently, other progenitors have emerged such as (c) the α6*β*4-double positive cells that can give rise to both alveolar and bronchiolar progenitors, (d) the basal-cell-“like” pods giving rise to variant Clara cells and AECII, and (e) the BASC also giving rise to variant Clara cells and AECII.

The adult lung epithelium has a remarkably low cell-turnover rate [84]. However, many injury models have been devised to selectively target various cell types in the lung epithelium. Upon injury, progenitor/stem cells, located in certain niches, undergo rounds of proliferation and differentiation to ensure epithelial regeneration.  Figure 9 summarizes the different adult epithelial progenitor cells that have unipotent or multipotent lineage-commitment capabilities upon injury.

Most of our knowledge regarding the lineage tree of epithelial cells derives from* ex vivo* studies (e.g., rat tracheal xenograft model) [85],* in vitro* cell cultures, and epithelial regeneration after injury. The latter includes the naphthalene injury model (to study regeneration of Clara cells), injury by inhalation of oxidants such as NO_2_/ozone and oxygen (to study regeneration of ciliated and AECI, resp.), and injury by inhalation of sulfur dioxide (SO_2_) (to study regeneration of multiple epithelial lineages). These experimental approaches are reviewed in [86].

Using the aforementioned approaches, it has been shown that in the upper (or proximal) airways, there are at least three distinct populations of epithelial progenitors. These include basal cells (Krt5^+^, Krt14^+^) [87–89] and variant Clara cells (Scgb1a1^+^, Cyp2f2^−^) within neuroepithelial bodies (as progenitors for ciliated and secretory cells) [90] as well as submucosal gland (SMG) duct cells (Krt14^+^) (as progenitors for the tracheal epithelium) [91]. As for the lower (or distal) airways, variant Clara cells at the bronchoalveolar duct junctions (BADJs) (Scgb1a1^+^, Sftpc^+^) have been suggested as stem cells (termed BASCs for bronchoalveolar stem cells) as they are capable of regenerating bronchiolar and alveolar cells [92, 93]. Moreover, AECII (Sftpc^+^) have been shown to be progenitors for AECI (Aqp5^+^) [94–96] and have recently been shown to be stem cells in the adult lung [74]. In addition, in the context of bleomycin injury, prelabeled Scgb1a1^+^ cells were shown to give rise to AECII in the fibrotic regions [74].

In a similar context, Kim et al. have identified BASCs as Cd45^−^, Cd31^−^, Sca-1^+^, and Cd34^+^ cells. These cells showed resistance to bronchiolar and alveolar damage and proliferated during epithelial regeneration following injury [93]. On the other hand, McQualter et al. were able to define lung epithelial stem/progenitor cells as Epcam^hi^, Cd49f^+^, Cd104^+^, and Cd24^low^. Their study was based on the ability of this cell population to form colonies in the presence of mesenchymal cells, giving rise to airway, alveolar, and mixed epithelial lineages. These epithelial stem/progenitor cells represent a minor fraction of the C57BL/6 adult lung epithelium [32]. This work was complemented by Chapman et al. who used the bleomycin injury model to demonstrate that Sftpc^−^, Cd49f^+^, and Cd104^+^ (α6*β*4-double positive) cells constitute a stable progenitor population that replenishes AECII during lung repair [31].

The lineage-tracing experiments described above, mostly done in the context of injury, allowed the identification of multipotent epithelial stem/progenitor cell populations along the proximodistal axis of the lung. However, during development and homeostasis, it appears that the epithelial stem/progenitor cells are mostly unipotent, giving rise only to one specific lineage. It has been described that both Clara cells and AECII are capable of long-term self-renewal, as the number of labeled cells did not decrease over time. For the other epithelial cell populations (AECI, ciliated cells, goblet cells, and neuroendocrine cells), it appears that not all the cells have long-term renewal capability as the number of labeled cells decreases over time. However, some of the labeled cells, like in the case of the ciliated cells, must have progenitor characteristics as a significant proportion of labeled cells is still observed 18 months after the initial labeling. It is therefore likely that the labeled populations are heterogeneous and will require further characterization. We refer the readers to a recent review by [97] for a complete description of lung epithelial stem/progenitor cells.

### 2.2. Mesenchymal Stromal (Stem) Cells (MSCs) in the Adult Mouse Lung

Contrary to the lung epithelium, the lineage tree of the lung mesenchyme is poorly understood. Although epithelial progenitors have been identified using the aforementioned epithelial injury models, very little success has been made in understanding the hierarchy, if any, of mesenchymal progenitors in the embryonic and adult mouse lungs.

**Figure 10:**
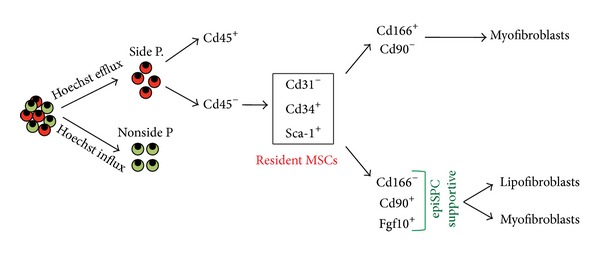
*Resident mesenchymal progenitor cells in the adult lung*. Cd45^−^ side population cells can be further characterized as Cd31^−^, Cd34^+^, and Sca-1^+^ mesenchymal cells. These cells were described as supporting the growth of epithelial stem/progenitor cells (epiSPC) that correspond to the α6*β*4-double positive cells. More recently, this subpopulation has also been subdivided into Cd166^+^, Cd90^−^ and Cd166^−^, and Cd90^+^ cells. The latter cells express* Fgf10*, support the growth of EpiSPC, and contain lipofibroblast progenitors.

Despite our poor knowledge regarding mesenchymal hierarchy in the lung, many studies have reported the presence of MSCs in adult mouse lungs and fetal human lungs [98, 99]. These cells have the ability to commit to multiple fates including chondrogenic, adipogenic, and myogenic lineages. Isolation and characterization of murine lung stem cells is based on their ability to efflux Hoechst 33342 dye and expression of stem cell antigen 1 (*Sca-1*) and mast/stem cell growth factor receptor (*Scfr* or* Kit)*. Cells that have the preference to efflux Hoechst 33342 dye are referred to as side population (SP) cells and they can be further categorized as having hematopoietic (Cd45^+^) or nonhematopoietic (Cd45^−^) origins [100, 101]. Cd45^−^, Cd31^−^, Sca-1^+^, and Cd34^+^ SP cells were found to highly express stem cell markers such as* Sca-1* and markers specific to mesenchymal lineages such as thymocyte antigen 1* (Thy-1 *or* Cd90)* and* Pdgfra*. This population of cells is referred to as resident MSCs and accounts for approximately 1% of the total C57BL/6 adult lung [102] (Figure 10).

Recently, it was shown that Cd45^−^, Cd31^−^, Epcam^−^, and Sca-1^+^ resident MSCs could be further refined into 2 main populations, Cd166^+^, Cd90^−^ and Cd166^−^, and Cd90^+^ [103]. It was shown that the Cd166^−^, Cd90^+^ population contains undifferentiated mesenchymal progenitors that are able to differentiate along the LIF and MYF lineages while the Cd166^+^, Cd90^−^ population contains more differentiated mesenchymal cells already committed to the MYF lineage. Interestingly, the Cd166^−^, Cd90^+^ cells have been shown to enhance the growth of epithelial stem cells (α6*β*4-double positive epithelial cells that are progenitors for both Clara cells and AECII) in coculture experiments compared to Cd166^+^, Cd90^−^ cells. In addition,* Fgf10* expression is found at high levels in Cd166^−^, Cd90^+^ cells and could be instrumental to support the growth of epithelial stem cells. It has also been shown that the epithelial-stem-cell-growth-supportive potential is severely reduced when these Cd45^−^, Cd31^−^, Epcam^−^, and Sca-1^+^ cells are grown* in vitro* and that this is associated with the loss of* Fgf10 *expression. In addition, culturing these cells with SB431542, a potent inhibitor of activin receptor-like kinase (Alk) receptors, which include Alk5 (also known as Tgf*β*r1), restores their epithelial-supportive potential. Transcriptomic analysis of Cd45^−^, Cd31^−^, Epcam^−^, and Sca-1^+^ cells grown* in vitro*, in presence or absence of SB431542, indicates the upregulation of several cytokines and growth factors in supportive resident MSC, including* Fgf10, Bmp3*, and insulin-like growth factor binding protein 2, 3, and 4 (*Igfbp2-4*). Interestingly, these growth factors are mostly linked to the LIF phenotype. It has been suggested that LIF could represent a niche for AECII [74]. In the future, it will be interesting to compare the epithelial-stem-cell-growth-supportive potential of Cd166^−^, Cd90^+^ cells differentiated towards the LIF lineage versus the MYF lineage.

In a similar context, we have demonstrated that the number of* Fgf10*-positive cells is amplified near the conducting airways following naphthalene injury in mice [104]. We propose that* Fgf10*-positive cells are critical mediators of epithelial stem/progenitor cell-mediated repair in the adult lung.

Models of fibroblast hyperproliferative diseases such as asthma, pulmonary arterial hypertension, and lung fibrosis have been routinely used and many hypotheses have been suggested regarding the origin of overproliferating fibroblasts. For instance, the origin of collagen-secreting,* αSma*-positive activated MYF in lung fibrosis is still unknown. It is believed that they originate from resident fibroblasts [105, 106], bone-marrow-derived circulating fibrocytes [107, 108], or AECII via epithelial-to-mesenchymal transition (EMT) [109, 110]. However, due to the lack of cell-specific markers, these hypotheses remain controversial. Rock et al. have recently demonstrated that stromal cells, in bleomycin-induced pulmonary fibrosis in mice, exhibit heterogeneity and that pericyte-like cells undergo proliferation but fail to express high levels of the MYF marker (*αSma*). The authors also used an* Sftpc* ^*Cre-ERT2*^ knock-in line to demonstrate that EMT by AECII does not contribute to fibrotic foci [96].

An emerging mesenchymal cell type in the lung is the LIF that is believed to be critical not only for surfactant production but also for alveolar septation. It has been demonstrated that injury to AECII, caused by prematurity, barotrauma, hyperoxia, nicotine, or infections, disrupts paracrine Pthrp signaling leading to LIF-to-“activated MYF” transdifferentiation [111]. Furthermore, several lung injuries, including cigarette-smoke exposure, induce the transdifferentiation of LIF to αSma^+^ MYF* in vitro* [112, 113]. Such transdifferentiation can be prevented and reversed* in vitro* using Ppar*γ* agonists such as Rosiglitazone [112, 114]. However, it is still unclear whether such transdifferentiation occurs* in vivo*, in idiopathic pulmonary fibrosis (IPF), for example. It has been proposed that Tgf*β*1, produced either by suffering AEC or by macrophages, induces this transdifferentiation into αSma^+^ MYF. As lung fibrosis involves proliferation of the activated MYF, it is logical to conclude that MYF exists in two different configurations: the “synthetic” MYF that are highly proliferative, undifferentiated, and that deposit high ECM and the “contractile” MYF that are not proliferative and that express the terminal differentiation marker* αSma*. The role of Fgf and Wnt signaling pathways in regulating the proliferation/synthetic versus the differentiation/contractile phenotype is still unknown and will need further investigation. Interestingly, bleomycin-induced lung fibrosis in mice is reversible (unlike human IPF which is 100% fatal). This reversibility is likely to offer unique opportunities to lineage-trace the activated MYF and identify which cell types they will turn into. A key in the treatment of diagnosed fibrosis lies in the enhancement of this natural process, which occurs in healthy animals and is likely to be impaired in diseased animals. It will be interesting in the future to test the role of Fgf10 in either preventing or reversing LIF-to-MYF transdifferentiation.* Fgf10* overexpression has been shown to prevent bleomycin-induced fibrosis when expressed during the inflammatory phase or to accelerate fibrosis resolution when delivered during the fibrotic phase [115]. Fgf10 acts by preventing apoptosis of AECII, by decreasing* Tgfb1* transcriptional expression, and by recruiting *γ* *δ*T cells. It is not clear if Fgf10 is also capable of directly inhibiting Tgf*β*1 activity. Fgf10 may be a very important therapeutic tool, much more powerful than Pirfenidone, which is the only available treatment for fibrosis approved so far [116].

## 3. FGF10, Lung Homeostasis, and Injury/Repair

After birth,* Fgf10* transcripts are detected at considerable levels in the lung during the alveolar stage (P5-P30) and during adulthood. This implies that the role of this growth factor is not limited to embryonic life but rather extends to postnatal life. However, the role of Fgf10 in lung homeostasis during postnatal life is poorly understood.

Inactivation of Fgfr2b signaling prenatally—but not postnatally—leads to emphysema [117]. Yet, it has been suggested that Fgfr2b ligands are not needed for normal lung homeostasis; rather they are required for repair and restoring homeostasis after hyperoxia [118] and naphthalene injury [104]. In the latter study, it was demonstrated that* Fgf10* expression is induced in PBSMCs and that Fgf10/Fgfr2b signaling between PBSMCs and surviving variant Clara cells is critical for epithelial regeneration.

In the lung,* Fgf10* overexpression postnatally has shown to produce reversible adenomas in addition to enriching the lung with* Sftpc*-positive cells [119]. This is consistent with the role of Fgf10 during lung development in inducing epithelial cell migration/invasion [7] and maintaining distal epithelial progenitors [19, 120]. In humans, the presence of strong expression of* FGF10* and* FGFR2b* is useful in differentiating congenital cystic adenomatoid malformation of the lung from type I pleuropulmonary blastoma [121].

Studying the impact of reduced* Fgf10* expression during embryogenesis on organ function postnatally and the function of Fgf10 itself postnatally in mice is clinically relevant because many human disorders related to* FGF10 *mutations have been reported. Mutations in the human* FGF10* gene are associated with autosomal dominant aplasia of lacrimal and salivary glands (ALSG) [122, 123]. These individuals suffer from irritable eyes and dryness of the mouth. Another autosomal dominant anomaly caused by* FGF10* mutations is the lacrimo-auriculo-dento-digital (LADD) syndrome. This syndrome is characterized by aplasia, atresia, or hypoplasia of the lacrimal and salivary glands, cup-shaped ears, hearing loss, and dental and digital abnormalities [124]. Moreover, recent evidence suggests that* FGF10 *mutations are associated with autism spectrum disorder (ASD) [125]. ASD patients suffer from social and communicative difficulties. Interestingly, we have recently found that* Fgf10* is expressed in the adult mouse brain, implicating a role in the control of neurogenesis [126]. We further demonstrated that* Fgf10*-expressing tanycytes are progenitors in the adult mouse hypothalamus.* Fgf10*-positive cells divide late into postnatal life and can generate both astrocytes and neurons, demonstrating that adult hypothalamic neurogenesis occurs and that Fgf10 could play a role in appetite and energy balance [127]. Furthermore,* FGF10* haploinsufficiency has been shown to be associated with chronic obstructive pulmonary disease (COPD). Lung function parameters from these patients indicate COPD and the modest response to treatment confirms irreversibility of the disease [128]. In addition,* FGF10* deficiency has been associated with BPD in humans [129]. It remains to be identified whether the impact of* FGF10* decrease affects only embryonic lung development and/or whether this growth factor plays also a functional role in postnatal lungs. The use of* Fgf10* conditional KO mice and transgenic mice to induce the expression of the soluble decoy* Fgfr2b*, to block all Fgf2b ligands, including Fgf10, in the context of normal development and injury will allow to test all these possibilities.

In conclusion, almost 20 years after the discovery of Fgf10, the characterization of its biological activities and its mechanism(s) of action during both embryonic and postnatal stages is still ongoing. The development of new* in vivo* tools to follow the fate of* Fgf10*-positive cells, to conditionally delete* Fgf10* expression and generation of specific* in vivo* reporter mouse lines to follow* Fgf10* activation will open the way to a better understanding of the role(s) of* Fgf10* in normal development and in the context of disease progression. In the future, the use of FGF10 recombinant protein to treat different pathologies in humans such as lung fibrosis is promising. Improving the stability of the protein and designing better approaches to gently deliver FGF10 locally in the lung will be the key for the successful use of FGF10 in humans.
